# New horizons in the compression of functional decline

**DOI:** 10.1093/ageing/afy145

**Published:** 2018-08-25

**Authors:** Peter G Gore, Andrew Kingston, Garth R Johnson, Thomas B L Kirkwood, Carol Jagger

**Affiliations:** 1 Newcastle University Institute for Ageing, Newcastle University, UK; 2 Newcastle University, UK; 3 Institute of Health & Society, Newcastle University, UK; 4 University of Copenhagen Center for Healthy Aging, Copenhagen, Denmark

**Keywords:** disability, function, disablement process, health ageing, older people

## Abstract

Population ageing, which has come about through the combination of increases in life expectancy, larger post-war cohorts reaching older age and reductions in fertility, is challenging societies and particularly health and care providers, worldwide. In Europe, the USA and Japan, there have been increases in years spent with disability and dependency. The majority of such research, as well as professional health and social care practice, measures loss of functional capability or need for social care, by aggregate disability scores, based around activities of daily living and instrumental activities of daily living. Although useful for defining whether an individual has passed a threshold, aggregate scores obscure how functional decline unfolds, and therefore where early intervention might improve intrinsic capacity and reverse or slow down decline, or maintain function. We propose a framework, the compression of functional decline (CFD), based on the latest understanding of the hierarchy of age-related functional decline, which has the potential to (i) help people understand how to live better for longer, (ii) allow the various stakeholders to be able to measure, at a population level, whether that is happening and (iii) identify which interventions are most effective at which stages. CFD is coherent with the World Health Organisation’s Healthy Ageing model and is more easily understood by stakeholders and older people themselves, than current indicators such as frailty. CFD thus provides a realistic view of age-related functional decline in the context of modifiable behaviour to counter widespread public misconceptions about ageing and inform improvements.

## Introduction

The world’s population is ageing, with the fastest growing section being those aged 80 years and over. In 1950 those ≥80 years comprised 7% of those aged 60 years and over, rising to 14% by 2013. Projections suggest that this proportion will rise further to 19% by 2050 and 28% by 2100 [1]. Moreover by 2050, there will be over twice as many people aged 80 and over in less developed countries compared with more developed ones, due to the faster population ageing taking place in the former. In many cases, this will mean an increase in the prevalence of chronic diseases, for which age is the over-riding risk factor. Nevertheless, population ageing does not necessarily result in greater prevalence of all diseases, and there is evidence from a number of countries that the prevalence and incidence of dementia and the years spent with cognitive impairment have reduced in the recent past, even though absolute numbers with these conditions have still increased [2, 3].

The relationship between morbidity and longevity was debated over 30 years ago and three theories emerged: compression of morbidity (COM) [4], expansion of morbidity (EOM) [5] and dynamic equilibrium (DE) [6]. If morbidity is defined in terms of major disease, most evidence points towards an EOM [7], with the interesting exception of cognitive impairment or dementia which shows a COM, the number of years with cognitive impairment having declined significantly between 1990 and 2011, at least for women [8]. These theories have fuelled the growing emphasis from extending length of life to maximising healthy years, or ‘health span’, the latter term now widely adopted by the biomedical community. Nevertheless, measuring ageing with a view to optimising ‘healthy ageing’ has been approached in many ways. Most recently, the World Health Organization has reflected the importance of functional ability by defining healthy ageing as ‘the process of developing and maintaining the functional ability that enables well-being in old age’ [9]. Indeed disability and dependency are feared most by older people, even more than death itself [10]. Moreover, at a population level, a number of countries are experiencing an expansion of disability and dependency [8, 11], though, with the increasing numbers of very old people, the picture may be becoming more complex, with compression and expansion existing in the same population but for different age-groups [12]. Therefore, understanding how age-related functional ability declines at an individual and a population level, and when and where to intervene to achieve compression, is important for both policy-makers and older people, to plan for and shape the future.

## The hierarchy of functional decline and compression

Over the subsequent half-century since the first development of activities of daily living (ADL) and instrumental activities of daily living (IADL) [13, 14], a variety of disability scales have been developed, including the Barthel scale [15] and the Groningen Activity Restriction Scale (GARS) [16]. These scales are used in research as well as clinical and professional practice, and most involving summing the number of activities/scores requiring help or that can only be performed with difficulty. Since early declines in function need not lead to the complete loss of an ADL/IADL, later developments have shown that it can also be important to take account of behavioural adaptations, for example taking more time to do a task or doing it less frequently, though recent research suggests that these adaptations do not always precede difficulty [17]. Issues with any scale that simply sums activities to form an overall disability score are that it (i) suppresses valuable information on the order and timing in which individual activity loss accumulates, (ii) does not identify the specific ADL/IADL deficits and (iii) sometimes includes only a small number of ADL/IADL, thereby reducing its sensitivity. Underlying causes of decline such as injury, disease and sarcopenia are associated with varying combinations of lower and upper body strength, balance, flexibility and manual dexterity, which can often be determined from the specific ADL/IADL. Thus, a simple score may obscure the appropriate interventions.

In fact, ADL/IADL appear to be lost in a particular order and this has been demonstrated by a number of cross-sectional and longitudinal studies, and across different age-groups, genders and populations [18–21]. Loss of function with age that follows this pattern can be described as ‘age-related’ to distinguish it from other patterns of progressive disability which are driven by causes other than age. This distinction is important since disability arising from other causes may follow very different patterns and require different approaches to intervention. A significant advantage of identifying an age-related hierarchy of loss is that it supports the design of services targeted at the *stages* of progress of disability, rather than at specific ages. The wide range of age at disability onset has been demonstrated with studies in Australian women (mean age at disability onset 79 years, range 45–88 years) [22], and in a British birth cohort where functional decline occurred as early as 43 years [21]. Given that individuals vary greatly in the time course of their ageing, the targeting of different stages in the hierarchy is likely to be both more successful and more cost-effective, as well as less ageist.

As longitudinal studies matured and accrued longer, and sometimes more frequent, follow-up, researchers heeded the call to better understand the progression of disability through the characterisation of trajectories [23]. Such trajectories, using summed disability scores, show remarkable similarities in terms of substantial numbers who either remain disability-free or experience catastrophic decline in function close to death [24, 25]. However, few have indicated timings, and none in a manner that could be used to target interventions.

**Figure 1.**
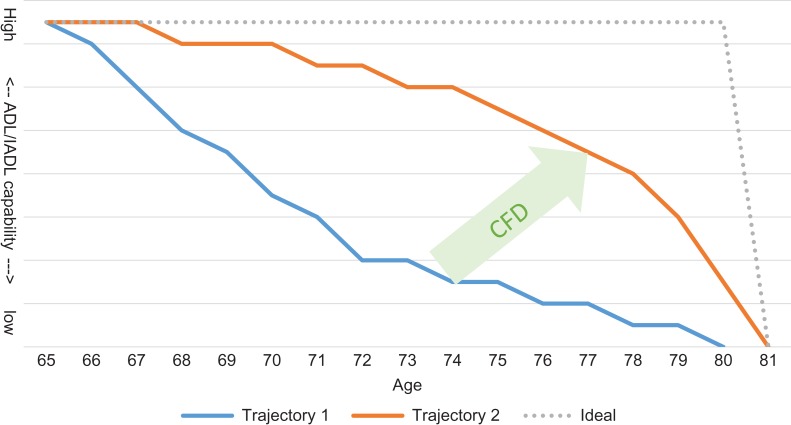
Hypothetical model of CFD where the goal is to shift trajectory 1 toward the ideal, resulting in trajectory 2 where higher levels of capability are maintained for longer *but without adding significantly to life expectancy*. (ADL/IADL capability is represented by individual activities in the order in which they are lost. The lowest point on the *Y*-axis is the point at which all 15 essential ADL/IADL are lost and dependency is reached.)

We propose representing trajectories in terms of the hierarchy of loss of activities with the time between stages (Figure 1) and we call this the compression of functional decline (CFD). Thus in Figure 1, rather than a composite disability score on the vertical axis, we have the individual activities in their hierarchical order of loss. The trajectory, therefore, represents the stages through which an individual goes as they progress through the disablement process encountering difficulty with, or inability to perform independently, each activity. In this framework, different diseases or health behaviours may impact at different stages, thereby leading to different trajectories, and there is evidence that better healthy behaviours can compress disability under certain conditions and potentially result in more rectangular trajectories [26]. We believe that strategies to compress the declines in ADL/IADL into the shortest possible period will help to focus policy on improving health span beyond the management of disease. CFD thus serves as a framework: (i) for quantifying decline and (ii) as a basis for a measurable and targeted strategy for prevention and early intervention that would engage individuals and their families as well as informing a wider group of stakeholders. Our experience to date shows it to be well understood by older adults, by their families and practitioners, and by businesses and policy-makers.

## Intervening to compress functional decline

The conceptual approach to intervening optimally to arrest functional decline is, we suggest, in the following four stages which should be undertaken in the given order:

- *Protection against decline*: Loss of muscle mass and function (sarcopenia) with age appears to be fairly consistent at approximately 1–2% per year past the age of 50 [27]. Although a number of studies have shown that decline in muscle strength with ageing contributes to functional limitations [28], muscle power declines earlier and more rapidly than strength, and, at least lower extremity muscle power, is a stronger predictor of functional performance at older ages than muscle strength [29]. There is emerging evidence that structured exercise of the correct type may delay the point at which functional loss occurs [30, 31]. The US National Institute on Aging further identifies balance and flexibility in their four necessary elements of exercise on its ‘Go4Life’ website: endurance, strength, balance and flexibility [32]. As the science is further developed we may be able to target better interventions to protect against decline.
- *Re-activation*: Recovering an ability such as walking 400 yds. Again, randomised controlled trial (RCT) has shown that targeted exercise can maintain this ability for longer, with no significant impact on mortality, thus suggesting that life expectancy free of mobility limitations may be increased [33].
- *Compensatory technology*: Regaining a lost ADL/IADL capability through the use of a minimally compensatory technology (e.g. a walking stick restoring the ability to walk). This is appropriate when an ADL/IADL ability is eventually lost (unavoidably or through a choice not to engage in re-activation). Assistive technology and home modification have been shown to reduce further functional decline in older adults with a disability [34], and to reduce the cost of care required by 3.8 hours per week [35].
- *Personal support*: Someone else undertakes the task for the older person (this indicates a loss of the ability, or replacement of a person’s own ability). This might be necessary at the most severe end of the hierarchy of loss of ability, but there are compelling reasons in the ‘use it or lose it’ vein to delay this stage for as long as possible, since even short-term muscle disuse accumulated over a period of time may contribute to the development of sarcopenia [36, 37]. Although this stage will not arrest further decline, it is important to include to demonstrate that earlier interventions might minimise the time in this stage.

## Benefits of CFD

The future will see higher absolute numbers of older people, and they will constitute a greater percentage of the population. Many societies are already seeing health and social care systems struggling to cope with their ageing populations. If the future sees further increases in years with high levels of dependency and poor levels of health, societies will face additional major—yet potentially avoidable challenges. The aim for ‘healthy ageing’ (or ageing well) is a good one at many levels, but the vision must be wider than the absence of disease and chronic conditions—it must include reduced time with functional loss i.e. compressed functional decline, allowing all the associated benefits of a more engaged and active older population. To achieve this, it must also drive changes in attitudes and individual behaviour, given their impact on trajectories [38].

Early intervention and prevention of decline is a sound idea, but how do we measure that in order to justify necessary investments, and how do we know we are not simply extending poor quality life? Given it is significantly affected by lifestyle choices, how do we communicate those choices to people, and then demonstrate what works, and what effect an individual’s choices are making? The gold standard of RCTs for evidence is both expensive, and arguably difficult to achieve in the community at the required scale—with significant ethical challenges for creating control groups—but we do need evidence. How can that be demonstrated?

CFD is an attempt to bring together evidence relating to how people functionally decline with age and time, into a simple framework that is understood and can be actioned by a wide range of stakeholders. This is complemented by an ordered, evidence-based intervention structure of delay, reactivate, compensate and then support with care. We have shown that there is good evidence that trajectories start at highly variable ages, and vary widely thereafter, but that they are also amenable to interventions [26]. Thus CFD will work both at the individual and population level.

While the evidence for how different (but statistically comparable) trajectories is at an early stage, we believe our CFD framework offers the basis for a new and useful dialogue around ageing well, among a wide range of stakeholders. Further, we suggest that as the measurement and understanding of trajectories grows, along with what can modify them, and the associated monetary, social and other costs, the CFD framework will provide a useful tool for innovators to assess and demonstrate their potential impact. By informing and shaping a compressed period of decline, the future could be one of more actively engaged older people for much longer, higher levels of quality of life, and significantly reduced health and social care and other costs for all stakeholders.

**Key points**

- The majority of research and practice measures functional decline by aggregate scores of activities of daily living.
- Aggregate disability scores obscure how decline progresses.
- We propose using the times between the hierarchy of activity loss to better quantify how to compress functional decline (CFD).
- CFD offers a framework for understanding the malleability of ageing in a way accessible to a wide audience including the public.
